# TRIM25 inhibits infectious bursal disease virus replication by targeting VP3 for ubiquitination and degradation

**DOI:** 10.1371/journal.ppat.1009900

**Published:** 2021-09-13

**Authors:** Suyan Wang, Mengmeng Yu, Aijing Liu, Yuanling Bao, Xiaole Qi, Li Gao, Yuntong Chen, Peng Liu, Yulong Wang, Lixiao Xing, Lingzhai Meng, Yu Zhang, Linjin Fan, Xinyi Li, Qing Pan, Yanping Zhang, Hongyu Cui, Kai Li, Changjun Liu, Xijun He, Yulong Gao, Xiaomei Wang

**Affiliations:** 1 Avian Immunosuppressive Diseases Division, State Key Laboratory of Veterinary Biotechnology, Harbin Veterinary Research Institute, The Chinese Academy of Agricultural Sciences, Harbin, PR China; 2 National Poultry Laboratory Animal Resource Center, Harbin, PR China; 3 Jiangsu Co-innovation Center for the Prevention and Control of Important Animal Infectious Disease and Zoonose, Yangzhou University, Yangzhou, PRChina; Loyola University Chicago, UNITED STATES

## Abstract

Infectious bursal disease virus (IBDV), a double-stranded RNA virus, causes immunosuppression and high mortality in 3–6-week-old chickens. Innate immune defense is a physical barrier to restrict viral replication. After viral infection, the host shows crucial defense responses, such as stimulation of antiviral effectors to restrict viral replication. Here, we conducted RNA-seq in avian cells infected by IBDV and identified TRIM25 as a host restriction factor. Specifically, TRIM25 deficiency dramatically increased viral yields, whereas overexpression of TRIM25 significantly inhibited IBDV replication. Immunoprecipitation assays indicated that TRIM25 only interacted with VP3 among all viral proteins, mediating its K27-linked polyubiquitination and subsequent proteasomal degradation. Moreover, the Lys854 residue of VP3 was identified as the key target site for the ubiquitination catalyzed by TRIM25. The ubiquitination site destroyed enhanced the replication ability of IBDV *in vitro* and *in vivo*. These findings demonstrated that TRIM25 inhibited IBDV replication by specifically ubiquitinating and degrading the structural protein VP3.

## Author summary

Owing to the immunosuppression and high mortality caused by infectious bursal disease virus (IBDV) in chickens, hosts urgently boost their antiviral immune responses to resist IBDV infection. Here, we identified the host antiviral factor TRIM25, which defended against IBDV infection by direct targeting the viral protein VP3 for subsequent ubiquitination and degradation. To our knowledge, this is the first report of host TRIM25 restricting IBDV replication. Additionally, we found that Lys854 of VP3 was the target site ubiquitinated by TRIM25. Mutation in the ubiquitination site of VP3 abrogated the degradation of VP3 and further enhanced IBDV replication, but did not influence its virulence. Our work not only uncovers an all-important molecular mechanism of chicken TRIM25 against IBDV but also provides new insights for developing high-titer live IBDV vaccines.

## Introduction

Infectious bursal disease (IBD) is an acute and highly contagious immunosuppression disease that occurs in 3–6-week-old chickens and is caused by infectious bursal disease virus (IBDV) [1,2]. IBDV infection results in high mortality, immunosuppression, and increased susceptibility to other pathogens [2,3]. IBDV is a non-enveloped, double-stranded RNA (dsRNA) virus belonging to the genus Avibirnavirus, family Birnaviridae [1,4–6]. The IBDV genome includes two segments, namely segment A (3.2 kb) and B (2.8 kb) [3,4]. Segment A, consisting of two overlapping open reading frames (ORFs), encodes the viral structural proteins VP2 and VP3 and the nonstructural proteins VP4 and VP5 [3–5]. Segment B contains a single ORF that encodes the viral RNA-dependent RNA polymerase (RdRP), VP1 [5]. Importantly, to assemble the intact IBDV particle, the main host-protective antigen VP2 forms the outer surface of the single layer viral capsid, and VP3 forms the inner surface of the viral capsid [5–11]. However, some researchers report that the capsid contains another polypeptide pVP2 [7]. Additionally, as a multifunctional structural protein, VP3 performs different functions in different replication stages [5,8–11]. VP3 is a major component of the IBDV ribonucleoprotein complex (RNP) alongside VP1 and dsRNA, facilitating the polymerase activity of VP1 [8,10]. In the assembly process, VP3 is responsible for the interaction with dsRNA, VP2, and VP1 to form intact viral particles, acting as a scaffolding protein [11]. Furthermore, VP3 can competitively combine dsRNA with MDA5 to escape the innate antiviral immunity of the host [9]. Collectively, VP3 is a crucial viral protein that facilitates IBDV replication by interacting with viral components or host factors in the life cycle of IBDV.

Virus infection is a continuous combat with the host [2,12]. During infection, the virus utilizes viral and host factors to facilitate its replication, whereas host conversely induces antiviral responses to resist virus infection [12,13]. As to the restriction of host to avian influenza virus (AIV) infection, the host primarily trigger innate immune responses by upregulating the expression of IFN-β and interferon-stimulated genes (ISGs) [14–17]. Additionally, the host observably increase the expression levels of type I, II and III IFN, IL-18, IL-4, and IL-13 during IBDV infection [5]. Furthermore, the host utilizes the interaction between host factors and viral proteins to inhibit IBDV replication [13,18,19]. For instance, nuclear factor 45 (NF45, also known as ILF2) is a negative regulator of IBDV replication through its interaction with VP1, VP2, and VP3 [18]. Eukaryotic translational initiation factor 4AII (eIF4AII) reduces IBDV replication by inhibiting the polymerase activity of VP1 [19]. Cyclophilin A interacts with VP4 and subsequently inhibits IBDV replication [13]. Identification of host antiviral factors can not only clarify the underlying mechanisms of host immune responses against IBDV infection but also be used as a strategy to modulate IBDV replication.

Tripartite motif (TRIM) proteins are found in most eukaryotes, including chickens, which contain a conserved structural N-terminal Really Interesting New Gene (RING) domain that enables TRIMs to function as ubiquitin E3 ligases and catalyze the ubiquitination of target proteins [20]. TRIMs are implicated in multiple cellular functions, ranging from transcriptional regulation to post-translational modifications (PTMs) [20]. Specifically, the most prominent role of TRIMs is to mediate antiviral activity [21–24]. A number of viral proteins are identified as the substrates of TRIMs during its antiviral process. TRIM32 senses and restricts IAV by ubiquitinating polymerase basic protein (PB1) [25]. TRIM69 restricts dengue virus (DENV) replication by ubiquitinating viral NS3 protein [26]. TRIM52 inhibits Japanese encephalitis virus (JEV) replication by ubiquitinating NS2A [27]. Similarly, chicken TRIMs also play an important role against viral infection. For example, TRIM25 inhibits avian leukosis virus subgroup A (ALV-A) replication through mediating the expression of type I interferon [28]. Another chicken TRIM protein, TRIM62, also mediates the replication of ALV and reticuloendotheliosis virus (REV) [29–30]. These results indicate that TRIM family proteins play a crucial role in resistance to viral infection owing to their ubiquitination activity.

Here, we identified TRIM25, through RNA-seq, as a host antiviral effector upregulated upon IBDV infection and found that TRIM25 significantly inhibited IBDV replication. Additionally, we demonstrated that TRIM25 interacted with VP3 of IBDV and subsequently induced its K27-linked ubiquitination and proteasomal degradation to suppress IBDV replication. Furthermore, we also identified Lys854 as the major ubiquitination site on IBDV VP3. When Lys854 was replaced with arginine, VP3 could not be ubiquitinated and degraded by TRIM25, and the replication ability of IBDV was enhanced *in vivo* and *in vitro*. This study revealed a restriction process of the host upon IBDV infection and elucidated the mechanism by which TRIM25 inhibits IBDV replication.

## Results

### TRIM25 is upregulated upon IBDV infection

**Fig 1:**
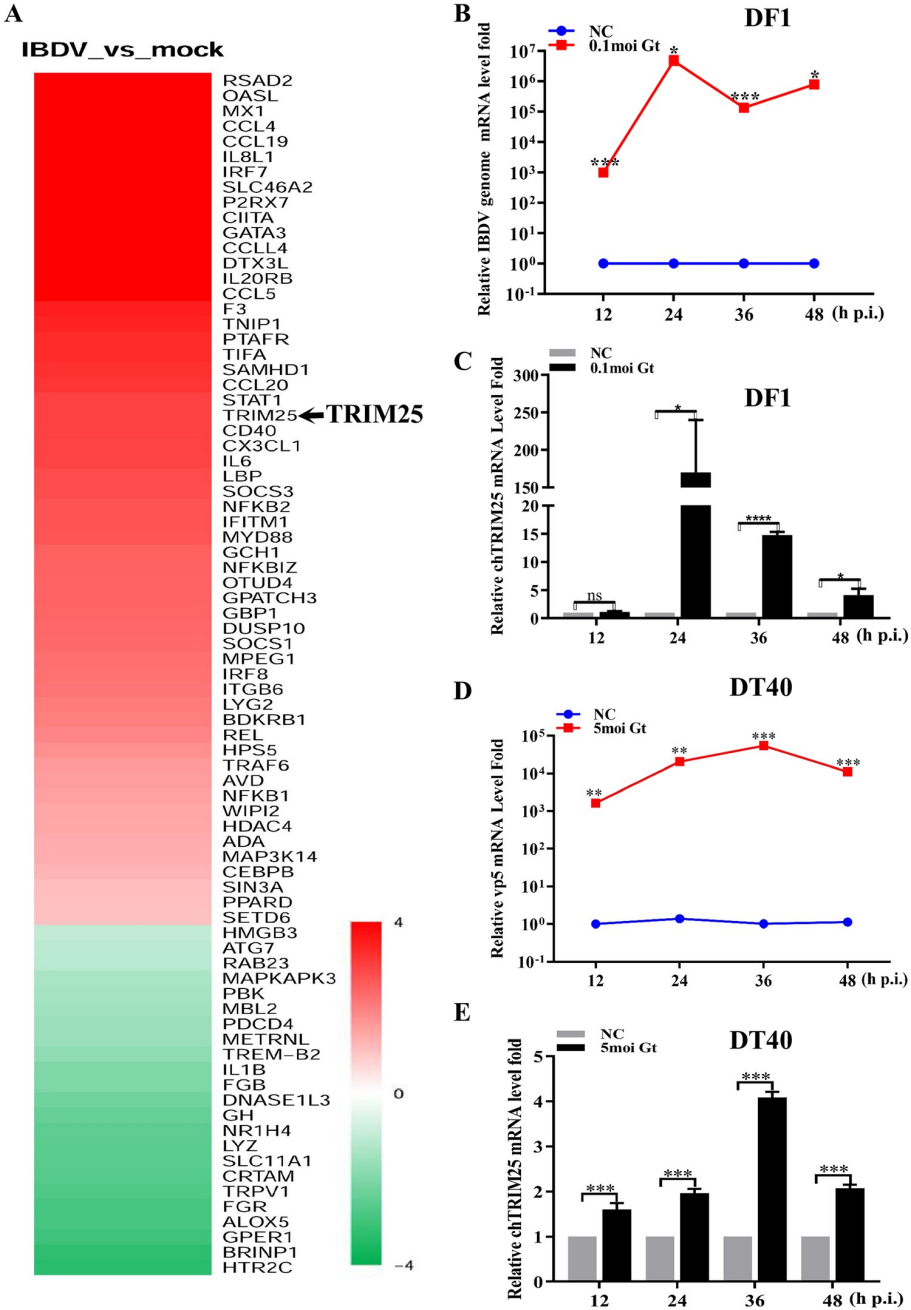
TRIM25 is upregulated upon IBDV infection. (A) The RNA-seq analysis of host molecules from host defense upon IBDV infection compared to that of non-infected in DF-1 cells at 24 h p.i. (B and C). IBDV infection causes the mRNA levels upregulation of TRIM25 in DF-1 cells. DF-1 cells were mock-infected or infected with IBDV Gt strain at an MOI of 0.1 and analyzed at 12, 24, 36, and 48 h p.i., respectively. (B) Relative fold-change of IBDV genome mRNA of IBDV of cell samples was quantified by RT-qPCR. (C) Relative expression of TRIM25 was quantified by RT-qPCR compared to the control. (D and E) IBDV infection causes upregulation of TRIM25 in DT40 cells. DT40 cells were mock-infected or infected with IBDV Gt strain at the MOI of 5 and analyzed at 12, 24, 36, and 48 h p.i., respectively. (D) The relative fold change of mRNA level of IBDV genome in cell samples were quantified by RT-qPCR. (E) Relative expression levels of TRIM25were quantified by RT-qPCR. All qPCR results are represented as relative fold changes normalized to β-actin controls. Three independent experiments were performed, and data are shown as mean ± standard deviations for triplicates from a representative experiment. *, *P* < 0.05; **, *P* < 0.01; ***, *P* < 0.001; ns, no significant difference.

To systematically investigate the potential host immune response to IBDV, we performed RNA-seq to screen out the host factors involved in IBDV infection 24 h post-infection (p.i.). As shown in Fig 1A, several host factors, particularly TRIM25, were upregulated upon IBDV infection. Previous studies showed that TRIM proteins play an important role in fighting viral infection [21–24,27–33]. To detect the relationship between TRIM25 and IBDV infection and the possible antiviral role of TRIM25, we infected DF-1 (immortal chicken embryo fibroblast) cells with the IBDV Gt strain at a multiplicity of infection (MOI) of 0.1 and DT40 (a chicken lymphoma cell line) cells at an MOI of 5. We then detected the mRNA levels of VP5 of IBDV in cell cultures and the endogenous expression level of TRIM25 via quantitative reverse transcription-PCR (RT-qPCR) at 12, 24, 36, and 48 h p.i., respectively. The RT-qPCR results showed that IBDV infected successfully, with relative mRNA levels of the IBDV genome increasing to 4.70×10^6^ in DF-1 cells (Fig 1B) and 5.44×10^4^ in DT40 cells (Fig 1D). TRIM25 expression levels were also significantly increased at different times, up to 169.9-fold in DF-1 cells (Fig 1C) and 4.1-fold in DT40 cells (Fig 1E) at 24 h p.i.. These results suggested that IBDV infection upregulated the expression of TRIM25 *in vitro*.

### TRIM25 overexpression inhibits IBDV replication

**Fig 2:**
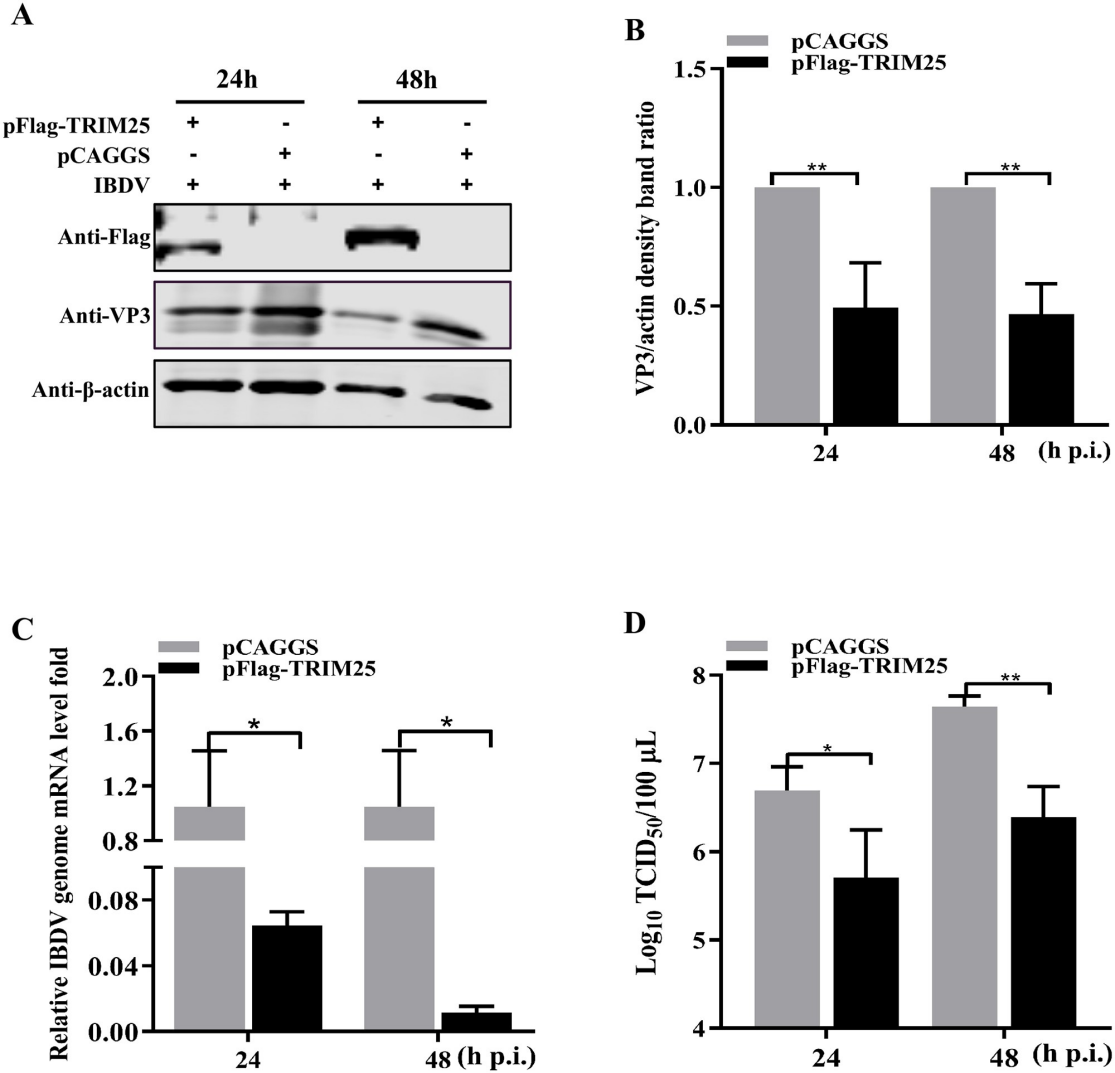
TRIM25 overexpression inhibits IBDV replication. (A-D) The influence of overexpression of pFlag-TRIM25 on IBDV replication. The DF-1 cells were transfected with 2μg pFlag-TRIM25 or pCAGGS at 24 h intervals and were infected with IBDV Gt strain at an MOI of 0.01. Subsequently, the cell and supernatant samples were collected at 24 and 48 h p.i, respectively. (A) Expression levels of TRIM25 and VP3 were determined by Western blotting. (B) Alternatively, relative intensities of VP3 were normalized to β-actin. (C) Relative fold-change of IBDV genome mRNA level of cell samples was quantified by RT-qPCR. (D) Released viral titer was detected and illustrated using a TCID_50_ assay. All qPCR results are represented as relative fold changes after being normalized to β-actin controls. The Western blotting results are representative of one of three independently performed. Three independent experiments were performed, and data are shown as mean ± standard deviations for triplicates from representative experiments. *, *P* < 0.05; **, *P* < 0.01.

To further evaluate TRIM25 overexpression on IBDV replication, DF-1 cells were infected with the IBDV Gt strain at an MOI of 0.01 at 24 h post-transfection (p.t.) with pFlag-TRIM25. Western blotting revealed that the overexpressed TRIM25 resulted in 2.02- and 2.14-fold decreases in the translation level of VP3, respectively (Fig 2A and 2B). Consistent with protein levels, RT-qPCR results showed that the levels of viral genome mRNA was decreased 16.28- and 89.83-fold in the robust expression of the pFlag-TRIM25 group (Fig 2C). Furthermore, the viral titer of the cell supernatant was detected with a median tissue culture infective dose (TCID_50_) assay at the same time point. The results showed that the released viral titers were decreased 14.79- and 15.85-fold following TRIM25 overexpression, respectively (Fig 2D). Altogether, these results indicated that the overexpressed TRIM25 could significantly inhibit IBDV replication.

### TRIM25 knockdown/knockout enhances IBDV replication

**Fig 3:**
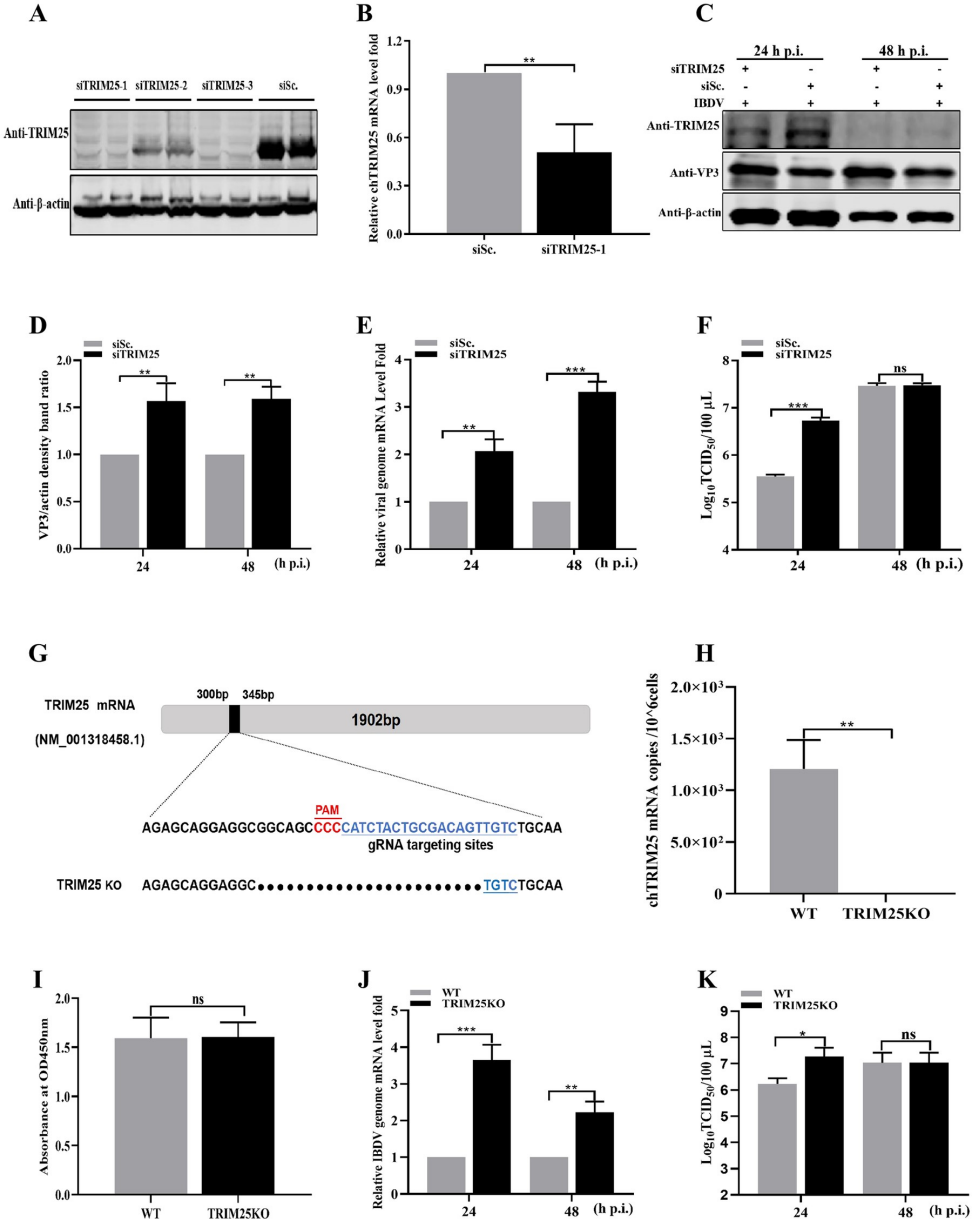
TRIM25 knockdown/knockout enhances IBDV replication. (A-B) Validation of the optimal siRNA targeting TRIM25 by Western blotting (A) and RT-qPCR (B). (A) HEK293T cells were co-transfected with siRNAi (siTRIM25-1, siTRIM25-2, siTRIM25-3, and negative siRNA control siSc.) and pFlag-TRIM25, and the expression level of TRIM25 was determined using Western blotting. (B) DF-1 cells were transfected with siTRIM25-1, and the expression levels of endogenous TRIM25 were detected by RT-qPCR. (C-F) Influence of knockdown of TRIM25 on IBDV replication. The DF-1 cells were transfected with 2μg siTRIM25-1 or negative siRNA control siSc. for 24 h and subsequently were infected with IBDV at an MOI of 0.01 for 24 and 48 h, respectively. (C) Expression levels of TRIM25 and VP3 were determined by Western blotting. (D) Relative intensities of VP3 were normalized to β-actin. (E) Relative IBDV genome mRNA level in cell samples was quantified by RT-qPCR. (F) Released viral titers were detected and illustrated using a TCID_50_ assay. (G-I) Construction of TRIM25KO DF-1 cell line. (G) Sequence analysis of WT and TRIM25KO DF-1cell lines. (H) TRIM25 mRNA level of WT and TRIM25KO DF-1 cell lines. (I) Cell viability of WT and TRIM25KO DF-1 cell lines. (J-K) Influence of knockout of TRIM25 on IBDV replication. The WT and TRIM25KO DF1 cells were infected with IBDV at an MOI of 0.01 for 24 and 48 h, respectively. (J) IBDV genome mRNA level in WT and TRIM25KO DF-1 cell lines. (K) TCID_50_ in WT and TRIM25KO cell lines. All the qPCR results are represented as relative fold-change after being normalized to β-actin controls. The Western blotting results were representative of one of three independently performed. Three independent experiments were performed, and data are shown as mean ± standard deviations for triplicates from representative experiments. *, *P* < 0.05; **, *P* < 0.01; ***, *P* < 0.001; ns, no significant difference.

To further determine the influence of TRIM25 knockdown on IBDV replication, we first selected three siRNAs targeting TRIM25 to evaluate knockdown efficiency. The Western blotting and RT-qPCR results indicated that siTRIM25-1 significantly reduced the expression level of TRIM25 (Fig 3A and 3B). Subsequently, DF-1 cells transfected with siTRIM25-1 for 24 h were infected with the IBDV Gt strain at an MOI of 0.01. The intracellular viral loads of cell cultures were detected by Western blotting and RT-qPCR at 24 and 48 h p.i., respectively. As shown in Fig 3C and 3D, siRNA-mediated knockdown of TRIM25 expression dramatically increased the expression levels of VP3. RT-qPCR results indicated that IBDV genome mRNA levels exhibited 2.11- and 3.32-fold increases, compared to the siSc. transfected control (Fig 3E). Furthermore, the result of TCID_50_ assay showed a 16.22-fold increase at 24 h p.i., compared to that in the siSc. transfected control (Fig 3F).

Additionally, to further verify the influence of TRIM25 on IBDV replication, the TRIM25KO DF-1 cell lines were constructed. Firstly, the sequence analysis of endogenous TRIM25 was detected to determine the success of the TRIM25KO DF-1 cell lines followed by the detection of TRIM25 mRNA levels and cell viability (Fig 3G–3I). The sequence analysis showed that the N-terminal of TRIM25 ORF has a 25 nucleotides deletion in the TRIM25KO DF-1 cell lines (Fig 3G). The result of RT-qPCR showed that the TRIM25 could not be detected in the TRIM25KO DF-1 cell lines (Fig 3H). CCK-8 assay results also indicated that the knockout of TRIM25 did not influence the cell viability of the cell line (Fig 3I). These findings demonstrated that the TRIM25KO DF-1 cell lines were constructed successfully. Subsequently, to verify the influence of TRIM25 knockout on IBDV replication, the wild-type (WT) and TRIM25KO DF-1 cell lines were infected at an MOI of 0.01 and collected for detection at 24 and 48 h p.i., respectively. RT-qPCR results showed that the TRIM25 knockout caused 3.65- and 2.22-fold increases of IBDV genome mRNA levels at 24 and 48 h p.i., respectively (Fig 3J). The released viral titer of supernatant showed a 14.67-fold increase at 24 h p.i., compared to that in the WT cell lines (Fig 3K). These results of overexpression, knockdown, and knockout demonstrated that TRIM25 could significantly inhibit IBDV replication.

### TRIM25 interacts with VP3

**Fig 4:**
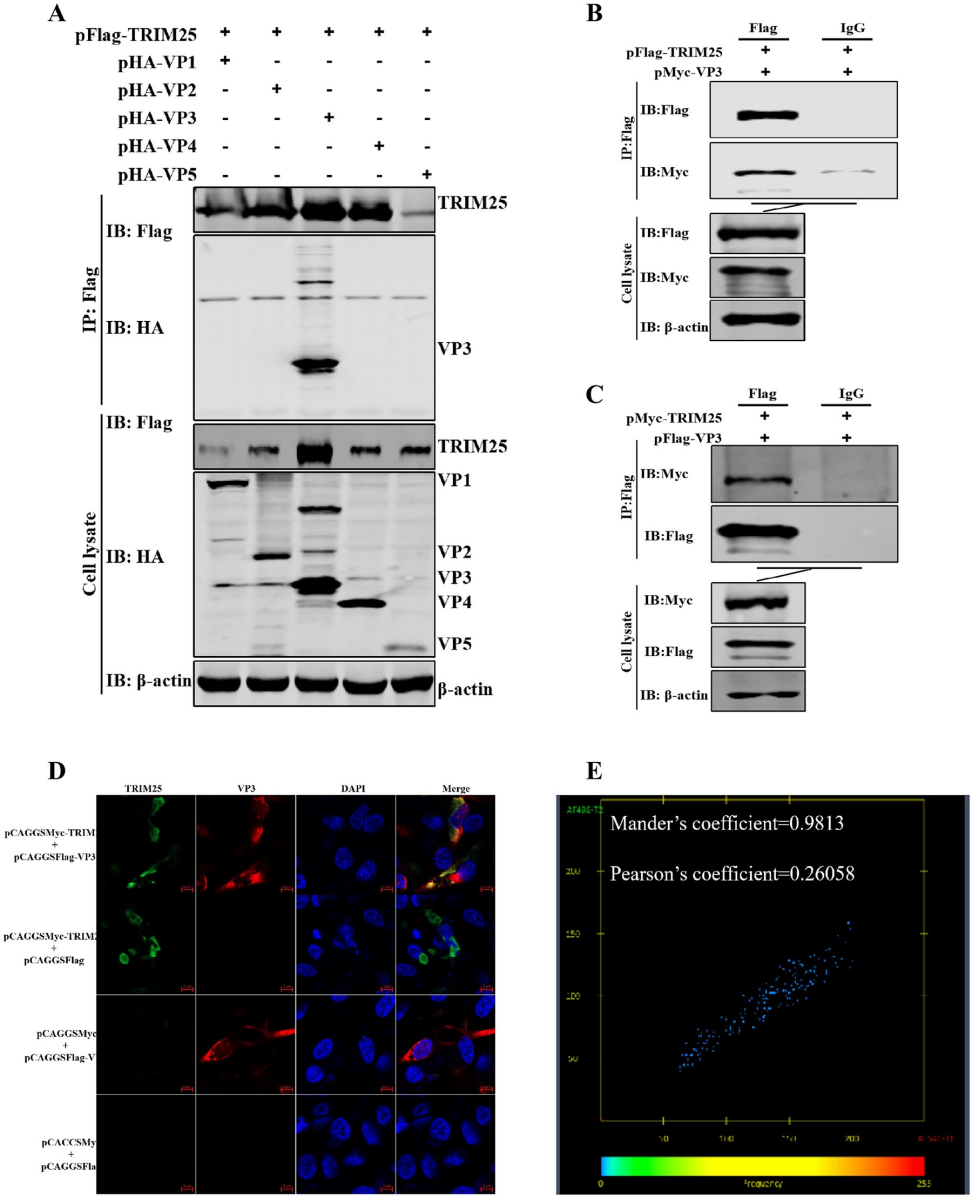
TRIM25 interacts with VP3. (A-C) Relationship between TRIM25 and VP3 was determined by Co-IP assays in DF-1 cells. (A) Lysates were incubated with anti-Flag mAb and tested using the indicated antibodies by Western blotting. Cells were co-transfected with pFlag-TRIM25 and pHA-VP1, pHA-VP2, pHA-VP3, pHA-VP4, or pHA-VP5 for 48 h, respectively. (B) Cells were harvested after co-transfection with pFlag-TRIM25 and pMyc-VP3 for 48 h. The lysates were incubated with 1 μg anti-Flag mAb or IgG produced in mice. (C) Cells were co-transfected with pFlag-VP3 and pMyc-TRIM25 at 48 h p.i. The lysates were incubated with 1 μg anti-Flag mAb or IgG produced in mice. (D) Confocal assays were used to assess the colocalization between TRIM25 and VP3. DF-1 cells were co-transfected with pFlag-TRIM25 and pHA-VP3 for 36 h. Cells were incubated with anti-Flag mAb produced in mice and anti-HA mAb produced in rabbits and the interaction between TRIM25 and VP3 was determined by confocal assay. (E) Colocalization analysis with Mander’s coefficient (0.9813) and Pearson’s coefficient (0.26058) on the confocal image.

To clarify how TRIM25 inhibits the replication of IBDV, we first tested whether TRIM25 interacts with viral proteins. We co-transfected the pFlag-TRIM25 plasmid with different viral proteins of IBDV plasmids (pHA-VP1, pHA-VP2, pHA-VP3, pHA-VP4, and pHA-VP5) into DF-1 cells for 36 h and detected the interaction via co-immunoprecipitation (Co-IP). Western blotting results showed that only VP3 could interact with TRIM25, while other viral proteins did not (Fig 4A–4C). Furthermore, confocal microscopy results indicated that TRIM25 was found in the cytoplasm and accumulated at the same position as VP3 (Fig 4D). The colocalization analysis showed that the Mander’s coefficient was 0.9813 and the Pearson’s coefficient was 0.26058 on the interaction between TRIM25 and VP3, indicating that the two proteins had a good interaction (Fig 4E). These results demonstrated that TRIM25 interacted with the structural protein VP3 of IBDV.

### TRIM25 promotes the degradation of VP3 in a proteasomal way

**Fig 5:**
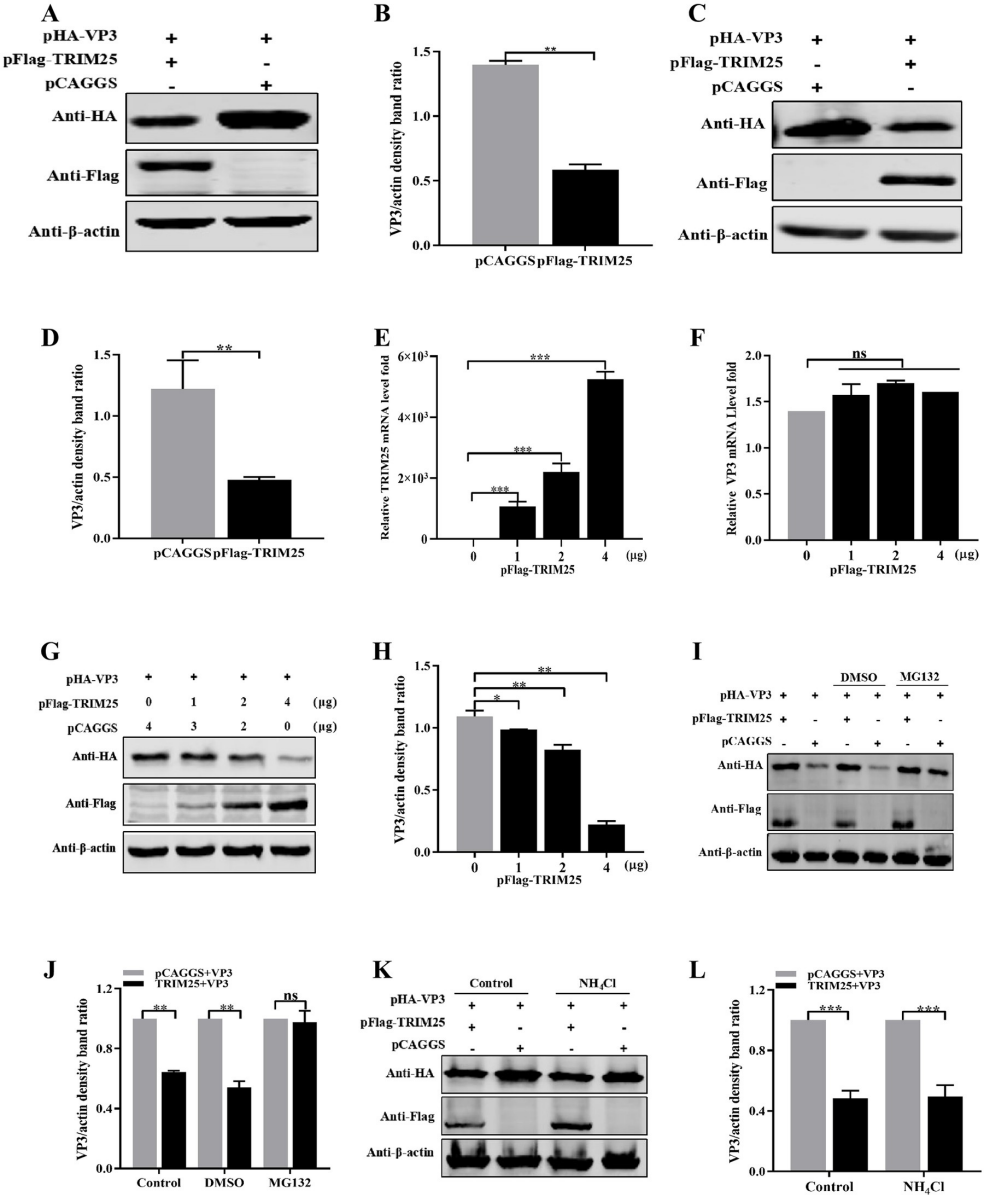
TRIM25 promotes the degradation of VP3 in a proteasomal way. (A-D) Exogenous expressed of VP3 was degraded by exogenously expressed TRIM25. (A and B) HEK293T cells were co-transfected with pHA-VP3 (0.5 μg) and pFlag-TRIM25 (4 μg) or empty vector (4 μg) for 36 h. (A) Degradation was shown by Western blotting with the indicated antibodies. (B) Relative intensities of VP3 were normalized to β-actin. (C and D) DF-1 cells were co-transfected with pHA-VP3 (0.5 μg) and pFlag-TRIM25 (4 μg) or empty vector (4 μg) for 36 h. (C) Expression levels of VP3 were determined by Western blotting using the indicated antibodies. (D) Relative intensities of VP3 were normalized to β-actin. (E-H) TRIM25 degraded the VP3 in a dose-dependent way. DF-1 cells were co-transfected with pHA-VP3 (0.5 μg) and increasing doses of pFlag-TRIM25 (0, 1, 2, and 4 μg) plasmids for 36 h. (E) TRIM25 mRNA levels were indicated by RT-qPCR. (F) VP3 mRNA level was indicated by RT-qPCR, and (G) Protein levels of VP3 was determined by Western blotting. (H) Relative intensities of VP3 were normalized with β-actin. (I and J) TRIM25 promoted VP3 degradation in a proteasomal way. DF-1 cells were co-transfected with pHA-VP3 (0.5 μg) and pFlag-TRIM25 (4 μg) or empty vector (4 μg) plasmids for 24 h, then treated with DMSO (negative control) or MG132 (10 μM). (I) Expression levels of VP3 was determined by Western blotting with indicated antibodies. (J) Relative intensities of VP3 were normalized with β-actin. (K and L) TRIM25 promoted VP3 degradation not depending on the lysosomal way. DF-1 cells were co-transfected with pHA-VP3 (0.5 μg) and pFlag-TRIM25 (4 μg) or empty vector (4 μg) plasmids for 24 h, then treated with PBS (negative control) or NH_4_Cl (20μM). (K) Expression levels of VP3 was determined by Western blotting with the indicated antibodies. (L) Relative intensities of VP3 were normalized with β-actin. All the qPCR results are represented as relative fold changes after being normalized to β-actin controls. Three independent experiments were performed, and data are shown as mean ± standard deviations for triplicates from a representative experiment. *, *P* < 0.05; **, *P* < 0.01; ***, *P* < 0.001; ns, no significant difference.

To further elucidate the mechanisms of TRIM25 inhibition of IBDV replication, we first examined whether TRIM25 could decrease the expression of VP3. TRIM25 and VP3 eukaryotic expression (pFlag-TRIM25 and pHA-VP3) plasmids were constructed and co-transfected into HEK293T cells. Western blotting results indicated that the expression of VP3 was significantly reduced after co-transfected with pFlag-TRIM25 (Fig 5A), reaching up to a 2.38-fold decrease (Fig 5B) compared to that in the pHA-VP3 only transfected group. To dotermine whether this decrease phenomenon existed in avian cells, the expression levels of pHA-VP3 co-transfected with pFlag-TRIM25were determined in DF-1 cells. Western blotting results showed that the expression levels of VP3 decreased 2.55-fold owing to the ectopic expression of TRIM25 in DF-1 cells (Fig 5C and 5D). These results demonstrated that TRIM25 could reduce the expression levels of VP3.

To further investigate whether TRIM25 degrades VP3 at transcriptional or translational level, pHA-VP3 plasmids were co-transfected with different doses (0, 1, 2, and 4 μg) pFlag-TRIM25 plasmid. RT-qPCR results showed that different doses TRIM25 were expressed successfully (Fig 5E), but did not influence the mRNA expression levels of *VP3* co-transfected (Fig 5F). While Western blotting results indicated that increasing amounts of TRIM25 resulted in 1.11-, 1.32-, and 4.92-fold decrease in the expression level of VP3. These results suggested that TRIM25 downregulated the translation levels of VP3 in a dose-dependent manner (Fig 5G and 5H).

The intracellular proteins were degraded primarily through the proteasomal or lysosomal pathways [34–36]. To determine the pathway through which TRIM25 degraded VP3, DF-1 cells were co-transfected with pFlag-TRIM25 and pHA-VP3 and treated with 10 nM proteasome inhibitor MG132 or 20 μM lysosome inhibitor NH_4_Cl at 24 h p.t. Western blotting results showed that the expression level of VP3 was not reduced in the group treated with MG132, compared to that in the DMSO-treated group, whereas NH_4_Cl did not influence the expression of VP3 (Fig 5I–5L). These results demonstrated that TRIM25 degraded VP3 through the proteasomal degradation pathway in a dose-dependent manner.

### TRIM25 induces the ubiquitination of VP3

**Fig 6:**
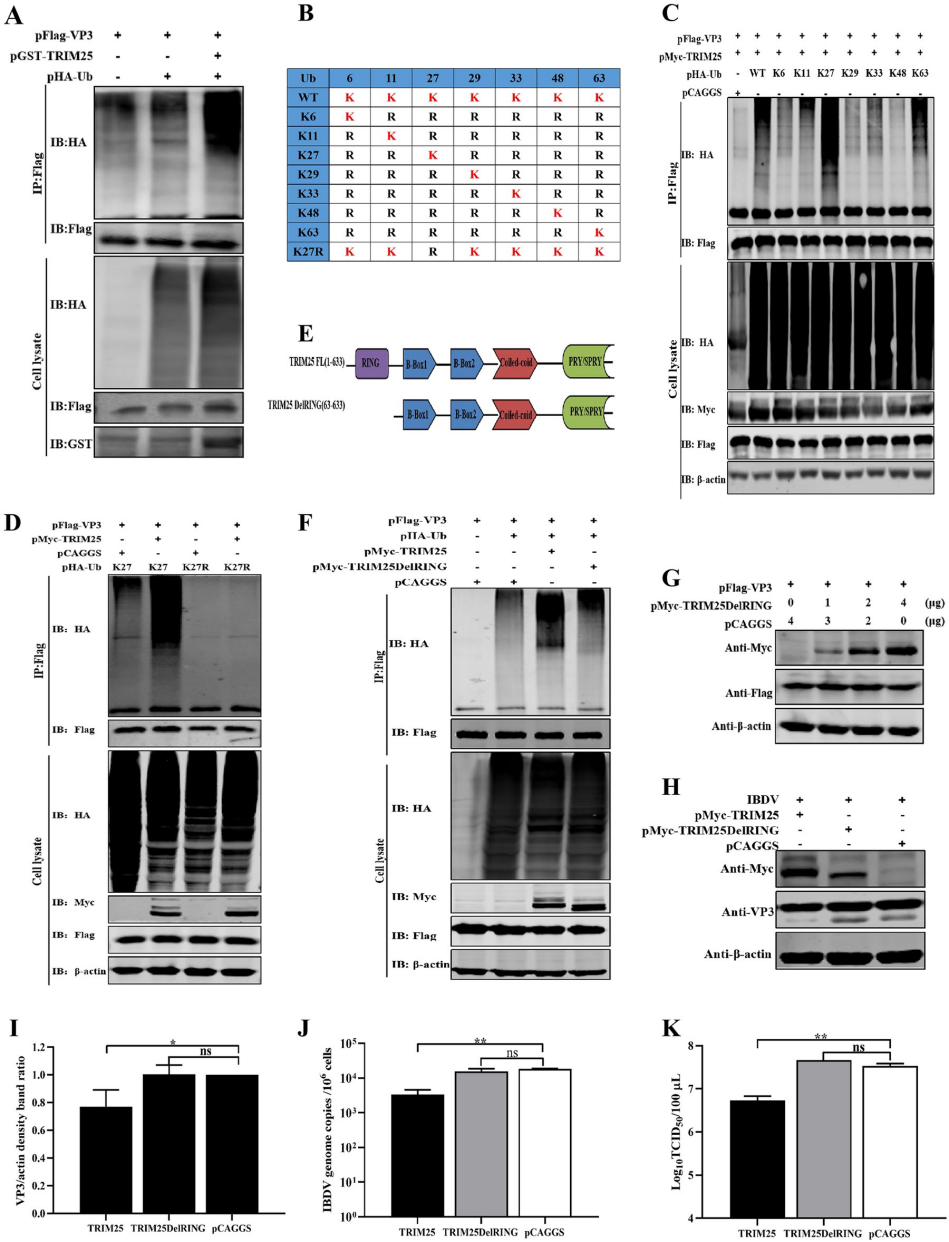
TRIM25 induces the ubiquitination of VP3. (A) TRIM25 promoted the ubiquitination of VP3. The pFlag-VP3 and pHA-Ub plasmids were co-transfected with or without pMycTRIM25 into HEK293T cells for 36 h. The lysate were incubated with indicated anti-Flag mAb and detected using an IP assay. (B) Schematic representation of the constructing strategy of eight Ub mutants (K6, K11, K27, K29, K33, K48, K63, and K27R). (C and D) TRIM25 catalyzed the K27-linked polyubiquitination of VP3. pFlag-VP3 and different pHA-Ub plasmids were co-transfected with pMyc-TRIM25 into HEK293T cells for 36h. Then, lysates were incubated with indicated anti-Flag mAb and detected using IP. (E) Schematic representation of the structure of TRIM25 and TRIM25DelRING. (F) Ubiquitination of VP3 catalyzed by TRIM25 depends on its E3 Ub ligase. HEK293T cells were co-transfected with pFlag-VP3 (0.5 μg) and pMyc-TRIM25 (2 μg) or TRIM25DelRING (2 μg) plasmids for the detection of VP3 ubiquuitination by immunoblotting. (G) Degradation of VP3 induced by TRIM25 depended on its E3 Ub ligase. HEK293T cells were co-transfected with pHA-VP3 (0.5 μg) and increasing doses of TRIM25DelRING (0, 1, 2, and 4 μg) plasmids for 36 h and further detected by Western blotting. (H-J) The DF-1 cells were transfected with 2μg pMyc-TRIM25, pMyc-TRIM25DelRING, or pCAGGS at 24 h intervals and were infected with IBDV Gt strain at an MOI of 0.01. Subsequently, the cell and supernatant samples were collected at 24h p.i, respectively. (H) Expression levels of VP3 was determined using Western blotting with indicated antibodies. (I) Relative intensities of VP3 were normalized with β-actin. (J) Relative fold change of IBDV genome mRNA level of cell samples was quantified by RT-qPCR. (K) Released viral titer was detected and illustrated using a TCID_50_ assay. All RT-qPCR results are represented as relative fold changes after being normalized to β-actin controls. Western blots were representative of one of three independently performed. Three independent experiments were performed, and data are shown as mean±standard deviations for triplicates from a representative experiment.*, *P* < 0.05; **, *P* < 0.01; ns, no significant difference.

Previous studies have demonstrated that the PTMs of proteins by ubiquitin (Ub) and their degradation by the Ub proteasome system (UPS) are the main regulatory processes during cellular life activities [12,34]. Hence, pFlag-VP3, pMyc-TRIM25 and pHA-Ub plasmids were co-transfected into HEK293T cells to examine the ubiquitination level of VP3. Western blotting results indicated that the overexpressed TRIM25 led to markedly increased ubiquitination of VP3, as determined via IP and ubiquitination assays (Fig 6A). To further confirm the type of polyubiquitination chains bound to VP3 catalyzed by TRIM25, the pHA-Ub WT and eight mutant Ub plasmids (K6, K11, K27, K29, K33, K48, K63, or K27R) were constructed and co-transfected with the pMyc-TRIM25 and pFlag-VP3 plasmids, respectively. IP and ubiquitination assay results showed that the ubiquitination levels of VP3 were increased by TRIM25 in the presence of K27 ubiquitin (Fig 6B and 6C). In contrast, the ubiquitination of VP3 was impaired when co-transfected with pHA-Ub (K27R) (Fig 6D). These results indicated that TRIM25 mediated the K27-linked polyubiquitination of VP3.

Besides, TRIM proteins contained a conserved structural arrangement of three N-terminal domains: RING domain, one or two B-boxes regions and a predicted coiled-coil region [24]. The RING domain of TRIMs is widely known to possess E3 Ub ligase activity in the ubiquitination process [12,24]. Therefore, TRIM25DelRING mutant (lacking the RING domain) plasmid was constructed to determine whether ubiquitination of VP3 depends on the Ub ligase activity of TRIM25. The results of IP and ubiquitination assay indicated that the ubiquitination levels of VP3 were significantly reduced in the presence of TRIM25DelRING (Fig 6E and 6F). These results demonstrated that the RING domain of chTRIM25 is critical for Ub ligase activity and VP3 ubiquitination.

Further experiments were performed to verify the importance of E3 ubiquitin ligase activity of TRIM25 in the degradation of VP3 and IBDV replication. Western blotting results showed that the expression levels of VP3 were not reduced co-transfected with different amounts of TRIM25DelRING plasmids (Fig 6G). Additionally, during IBDV replication, Western blotting results showed that overexpressed TRIM25DelRING did not reduced the expression level of viral protein (Fig 6H and 6I). Similarly, RT-qPCR and TCID_50_ results showed that the mRNA levels of IBDV genome and the viral titer of cell supernatant did not decreased in the presence of TRIM25DelRING, but did in the WT TRIM25 overexpression group (Fig 6J and 6K). These results demonstrated that the ubiquitin ligase activity of TRIM25 is critical for the degradation of the target protein VP3 and the antiviral activity against IBDV.

### Lys 854 of VP3 is a ubiquitination site of TRIM25

**Fig 7:**
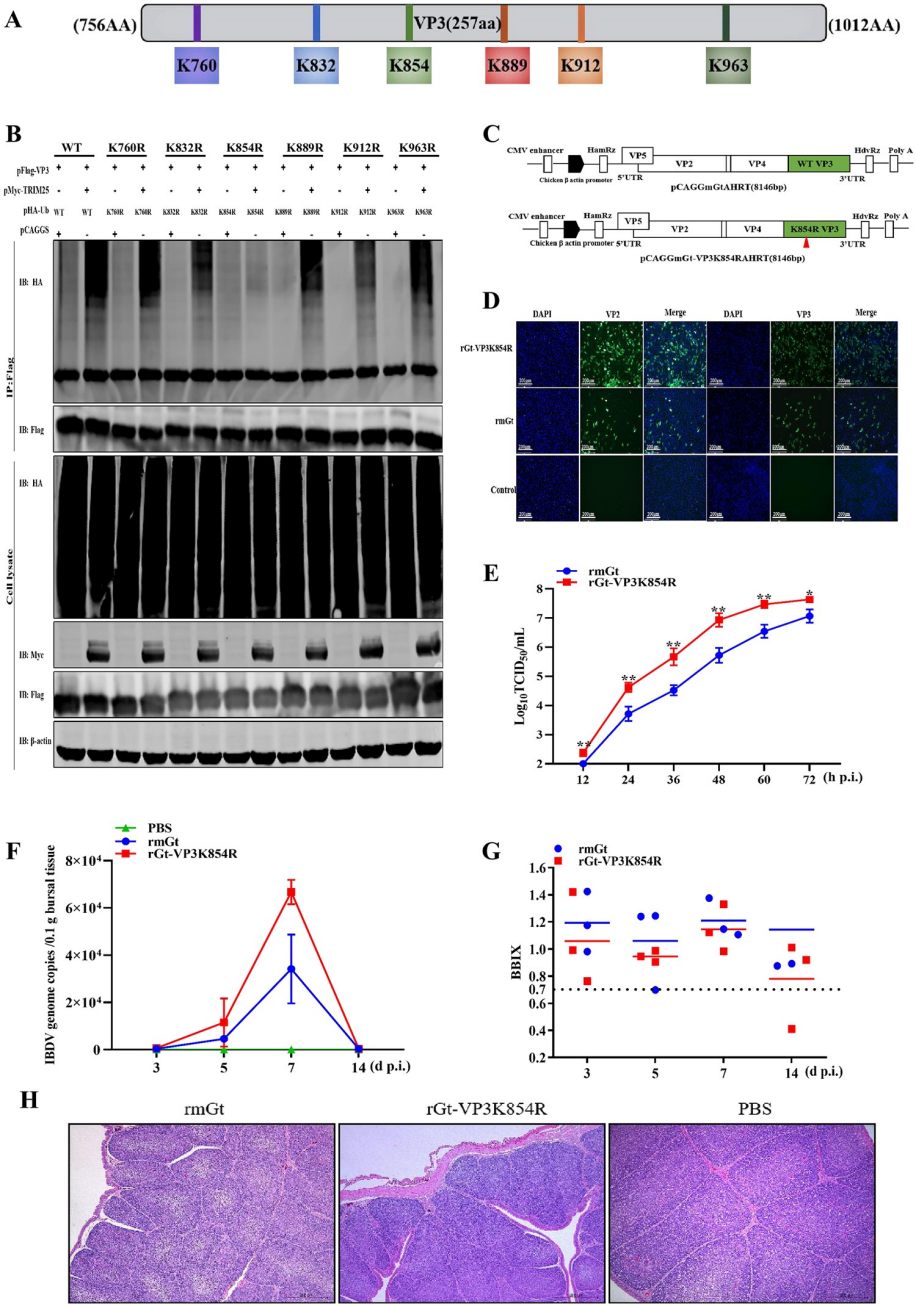
Lys854 of VP3 is a ubiquitination site of TRIM25. (A) Schematic representation of the possible ubiquitination site of VP3. (B) The amino acid of VP3 was ubiquitinated by TRIM25. Different VP3 plasmids (WT and different mutants) and pHA-Ub were co-transfected with pMyc-TRIM25 for 36 h. Then, the lysates were analyzed by immunoblotting. (C) Schematic representation of the strategy of rescuing IBDV. Segments A and B were constructed according to the instruction in the Materials and methods section. (D) IFA assay. DF-1 cells were infected with rmGt and rGt-VP3K854R viruses and were detected with anti-VP2/VP3 mAb. (E) Replication activity between rmGt and rGt-VP3K854R was detected using a TCID_50_ assay *in vitro*. CEFs were infected with WT and mutant IBDV at the MOI of 0.01 for 12, 24, 36, 48, 60, and 72 h. The 3-week-old SPF chickens were challenged with 10^5.8^TCID_50_/200 μL of two viruses, and bursae samples were collected at3, 5, 7, and 14 d p.i. (F) IBDV genome copies of rmGt and rGt-VP3K854R were detected by RT-qPCR *in vivo*. (G) BBIX of all challenged bursae. (H) Histopathological examination of bursae at 7 day p.c. Three independent experiments were performed, and data are shown as mean ± standard deviations for triplicates from a representative experiment. *, *P* < 0.05; **, *P* < 0.01.

Protein ubiquitination is a process by virtually transferring the ubiquitin molecules to lysine residues of the target protein [12]. As shown in Fig 7A, six potential ubiquitination sites (Lys760, Lys832, Lys854, Lys889, Lys912, and Lys963) of VP3 were predicted by the UbPred program (http://www.ubpred.org/) [26]. Different VP3 substitutions (pFlag-VP3K760R, K832R, K854R, K889R, K912R, or K963R) by individually replacing one of the six lysine residues with arginine, were constructed and co-transfected with pFlag-TRIM25 and pHA-Ub plasmids, respectively into HEK293T cells to detect the ubiquitination levels of VP3. Western blotting results showed that the ubiquitination of VP3K854R substitution could not be induced by TRIM25, while the ubiquitination of other five substitutions did (Fig 7B). These results indicated that Lys854 of VP3 was the key ubiquitination site of TRIM25.

### Destroy ubiquitination of VP3 enhances the replication ability of IBDV

To identify the importance of Lys854 of VP3 in the replication ability of IBDV, we first rescued WT IBDV (rmGt virus) and Lys854-mutant IBDV lacking the key ubiquitination site of VP3 (rGt-VP3K854R virus) using reverse genetics in chicken embryo fibroblasts (CEFs) [37] (Fig 7C). The indirect immunofluorescence assay (IFA) results showed a strong VP2 and VP3 signal, indicating that the two viruses were rescued successfully (Fig 7D). To further compare the replication abilities of rmGt and rGt-VP3K854R *in vitro*, the two viruses were inoculated into CEFs at an MOI of 0.01 and harvested at 12, 24, 36, 48, 60, and 72 h p.i. Compared to rmGt, the growth curve showed the released viral titers of rGt-VP3K854R with a 4–16-fold increase at 12–72 h p.i. (Fig 7E). In addition, we infected 3-week-old specific-pathogen-free (SPF) chickens with rGt-VP3K854R and rmGt virus at a dose of 10^5.8^TCID_50_/200 μL and collected bursae at 3, 5, 7, and 14 days p.i., respectively. RT-qPCR results indicated viral loads of rGt-VP3K854R, with a 2–3-fold increase in bursae (Fig 7F). These results suggested that the destruction of the Lys854 ubiquitination site of VP3 could enhance the replication abilities of IBDV *in vitro* and *in vivo*.

To further examine the influence of Lys854 of VP3 on the virulence of IBDV, the SPF chickens were infected with rGt-VP3K854R and rmGt viruses at a dose of 10^5.8^TCID_50_/200 μL, respectively. The bodies and bursae were weighed for the calculation of bursa: body weight index (BBIX) [38], and the bursa of Fabricius was fixed for histopathological study at different point times. Results showed that the BBIX of the rGt-VP3K854R group was above 0.7 at 3, 5, 7, and 14 days p.i., respectively, similar to that of rmGt (Fig 7G). Furthermore, the histopathological analysis results showed that rGt-VP3K854R did not induce any lesions in the bursae of SPF chickens, similar to rmGt (Fig 7H). These results demonstrated that the replacement of Lys854 with arginine in VP3 did not influence the virulence of IBDV (Fig 7H).

## Discussion

IBDV infection causes severe damage to the bursa of Fabricius of chickens and immunosuppression with compromised humoral and cellular immune responses, leading to susceptibility of chickens to other diseases. IBDV infection is actually a complex conflict between the host and virus. Therefore, understanding the interaction between IBDV and the host can not only clarify the infection process of virus in host cells but also help discover antiviral factors and reveal their specific antiviral mechanisms. In our study, TRIM25 was identified as a host restriction factor for IBDV. The results indicated that TRIM25 inhibited IBDV replication by directly interacting with the viral protein VP3 for ubiquitination and degradation. In addition, our results showed that Lys854 of VP3 was the key ubiquitination site. More importantly, the replacement of Lys854 with arginine destroyed the ubiquitination and degradation of VP3 and further enhanced the replication ability of IBDV *in vivo* and *in vitro*.

Numerous TRIM family proteins play important roles in inhibiting virus replication. Furthermore, the most common pattern of the regulatory role of TRIMs in viral replication is the feedback mechanism, namely, virus infection upregulates the mRNA expression levels of TRIMs, while host factors inhibit viral replication. For example, TRIM22 is upregulated during viral infection and significantly inhibits the replication of AIV [21]. Viral infection upregulates the expression of TRIM62, which plays important roles in inhibiting ALV [29] and REV replication [30]. In our study, RNA-seq results showed that the mRNA levels of four TRIMs (TRIM24, TRIM25, TRIM58 and TRIM71) were up-regulated by IBDV infection (S1 Fig). While only TRIM25 participated in the defense response of the host against viral infection according to Go functional analysis results (Fig 1A). Therefore, TRIM25 might be a candidate factor to restrict IBDV infection and merits further study.

Previous studies suggest that host factors regulate IBDV replication mainly through the following two ways. First, the host influences IBDV replication by upregulating or downregulating of the immune response. For example, glucocorticoid-induced leucine zipper (GILZ) interacts with VP4 to suppress IFN expression and enhance viral replication [39]. Casein kinase 1 alpha reduces the stability of IFNR by interacting with VP2 to promote IBDV replication [40]. Second, the host regulates IBDV replication through mediating the VP1 viral protein polymerase activity directly [18,41,42]. The voltage-dependent anion channel 1 (VDAC1) promotes the stability of the VP1 and VP3 complexes and subsequently increased VP1 polymerase activity to promote viral replication [41]. Besides, eIF4AII reduces the polymerase activity of VP1 by interacting with VP1 to inhibit virus replication [19]. While our study revealed a new regulatory pathway to limit IBDV infection, i.e., TRIM25-mediated direct targeting and degradation the viral protein VP3. Specially, TRIM family proteins exerted ubiquitous regulatory role by direct interacting with viral proteins [43,44]. In the regulation of single strand RNA virus replication, TRIM22 can direct reduce nucleoprotein (NP) protein expression levels to inhibit IAV replication [21], and TRIM69 targets NSP3 protein of the dengue virus for subsequent degradation to inhibit its replication [26]. Similarly, to regulate retrovirus replication, TRIM33 targets HIV integrase for degradation [45]. In addition, the targeted regulatory role of TRIMs is specific. In the regulatory process of IAV replication, TRIM32 direct targets PB1 for degradation, while TRIM35 direct interacts with PB2 [16,25]. Our results demonstrated that TRIM25 only interacted with the viral protein VP3 of IBDV but not with other IBDV proteins. The present study adds chicken TRIM25 to the expanding family of TRIM proteins that inhibit virus replication by targeting viral proteins.

TRIMs have garnered increasing attention for their ability to regulate viral replication through the PTMs of Ub [43,44]. TRIMs regulate virus replication by indirectly mediating the innate immune response or direct targeting viral proteins for ubiquitination [12,44,46]. TRIM25, as a TRIMs, inhibits the replication of various viruses, such as, IAV, porcine reproductive and respiratory syndrome (PRRSV) and vesicular stomatitis virus (VSV), mainly by promoting the ubiquitination of retinoic acid-inducible gene I (RIG-I) [47–50] to upregulate the innate immune response in mammalian cells. Similarly, TRIM25 can ubiquitinate the downstream molecules, mitochondrial antiviral-signaling protein (MAVS) or TNF receptor-associated factor 6 (TRAF6) of the RIG-I/melanoma differentiation-associated gene 5 (MDA5) signaling pathway to regulate Sendai virus (Sev) or IAV virus replication [50–52]. Even previous studies demonstrate that TRIMs direct regulate virus replication by interacting with viral proteins. Our results revealed, for the first time, that chicken TRIM25 could direct target and ubiquitinate VP3 for degradation to suppress IBDV replication in avian cells.

In mammals, the RING domain of TRIM25 is proved to be the primary functional ubiquitination domain [32,47]. Our studies demonstrated that the TRIM25DelRING mutant failed to catalyze the ubiquitination of VP3 and inhibit IBDV replication. These results showed that the RING domain of avian TRIM25 also played an important role in exerting E3 Ub ligase and antiviral activities as the RING domain in mammalian cells. TRIMs degrade proteins mainly relying on the Ub -proteasomal pathway [34]. Our results showed that MG132 greatly restored VP3 degradation induced by TRIM25 (Fig 5I), whereas NH_4_Cl had no apparent effect on the degradation of this protein (Fig 5K). These findings support the conclusion that TRIM25 induces VP3 degradation via the proteasomal pathway. Additionally, the UPS, the major intracellular pathway for protein degradation, is responsible for eliminating short-lived and soluble dysfunctional cell components to regulate various processes, such as protein homeostasis and translation [36]. Hence, we speculated that the degradation of viral proteins in the Ub proteasome pathway could be an important approach for host factors to restrict IBDV infection.

As a multifunctional structural protein related to IBDV replication, VP3 can not only bind to VP1 and dsRNA to form the ribonucleoprotein (RNP) complex to regulate viral RNA replication and translation but can act as a scaffolding protein to stabilize the structures of intact virions [8,9,53–57]. These researches mentioned above show that VP3 undergoes significant function with IBDV replication. Our results demonstrated that TRIM25 specifically targeted and degraded VP3 to inhibit IBDV replication, resulting in an eventual decrease in IBDV progeny viral titers. These findings indicated that TRIM25 induced the ubiquitination and degradation of VP3 (Figs 5A and 6A), which might damage the the formation of the RNP complex to inhibit IBDV replication. Hence, these results suggested that TRIM25 might restrict IBDV infection by bridging VP3 to affect multiple replication processes.

The ubiquitination process of TRIM25 is completed by transferring Ub molecules to a lysine residue of the target protein to form a polyubiquitin chain [54]. Our results indicated that Lys854 was the key ubiquitination site of VP3. When the key ubiquitination site was destroyed, the progeny virus titer of rGt-VP3K854R was 4–16-fold higher than that of rmGt *in vitro* (Fig 7E). Similarly, the IBDV genome copies of rGt-VP3K854R in SPF chickens were increased by 2–3 fold (Fig 7F). The results indicated that the mutation of ubiquitination site enhanced the replication ability of IBDV *in vivo* and *vitro*. The prevention and control for IBDV mainly rely on attenuated live vaccines, and these vaccines are usually produced by CEFs. Additionally, our animal experiments also found that IBDV mutants in the ubiquitination site of VP3 had no effect on its virulence. Therefore, the ubiquitination site of IBDV VP3 was considered a possible mutation strategy to rescue recombinant virus and subsequently improve the viral titer, providing new insights for the development of high-titer and low-cost IBDV vaccines.

In summary, our findings suggest a new role of chicken TRIM25 in the inhibition of IBDV replication via mediating VP3 K27-linked polyubiquitination and proteasomal degradation. In addition, we found that the Lys854 mutation disrupted the ubiquitination and degradation of VP3 and enhanced the replication ability of IBDV *in vitro* and *in vivo*. Collectively, our findings reveal an important molecular mechanism of TRIM25 against IBDV infection, which improves our understanding of host natural defense mechanism and facilitates the development of more effective strategies for controlling IBDV infections.

## Materials and methods

### Ethics statement

All experiments on WT (rmGt) and mutant IBDV (rGt-VP3K854R) viruses were conducted within the biosafety level 2 (P2+) facilities in the Harbin Veterinary Research Institute (HVRI) of the Chinese Academy of Agricultural Sciences (CAAS). This study was carried out in strict accordance with the recommendations in the Guide for the Care and Use of Laboratory Animals of the Ministry of Science and Technology of the People’s Republic of China.

### Cells and virus

The HEK293T cells and DF-1 cells were purchased from the ATCC and maintained in Dulbecco’s modified Eagle’s medium (DMEM; C11995500BT, Gibco, China) containing 10% fetal bovine serum (FBS; 10091–148, Gibco, New Zealand), penicillin, and streptomycin (15140–122, Gibco, USA). Trypsin-EDTA (0.25%; 1×; 25200–056, Gibco, Canada) was used. DT40 cells were obtained from Dr. Venugopal Nair of the Pirbright Institute and cultured in RPMI 1640 medium (C11875500BT, Gibco, USA) supplemented with 50μM β-mercaptoethanol, 1% L-glutamine (25030–081, Gibco, USA), 2% chicken serum (C5405-100ML, Sigma, GER), and 5% FBS. CEFs were prepared from 9-day-old embryos of SPF chicken. The 293T, CEF, and DT40 cells were maintained in a humidified incubator containing 5% CO_2_ at 37 °C. DF-1 cells were maintained in a humidified incubator containing 5% CO_2_ at 38.5 °C. The IBDV Gt strain was identified and preserved in our laboratory.

### Antibodies and reagents

Many antibodies were used in the experiments to detect protein expression and interaction. Mouse monoclonal anti-IBDV VP2 and VP3 antibodies were produced and preserved in our laboratory. Other antibodies used in our study were mouse anti-FLAG M2 (F1804, Sigma, USA), rabbit anti-TRIM25 antibody (produced by Genscript), mouse-anti-HA monoclonal (H9658, Sigma, USA), mouse anti-β-actin monoclonal (A1978, Sigma, USA), rabbit anti-Myc (M4439, Sigma, USA), goat anti-rabbit IgG H&L (Alexa Fluor 488; A-11008, Invitrogen, USA), goat anti-mouse IgG H&L (Alexa Fluor 546; A11003, Invitrogen, USA), goat anti-mouse IgG (whole molecule) FITC (F9137, Sigma, USA), IRDye 680RD goat anti-rabbit IgG H&L (926–68071, LiCor Bio-Sciences, USA), and IRDye 800CW goat anti-mouse (926–32210, LiCor Bio-Sciences, USA) or goat anti-rabbit IgG H&L (926–32211, LiCor Bio-Sciences, USA) antibodies. The proteasome inhibitor MG132 (HY-13259, MCE, USA) and lysosome inhibitor NH_4_Cl (A9434, Merck, USA) were used for the degradation assays. Protease inhibitors, 1mM phenylmethanesulfonyl fluoride (PMSF; ST506, Beyotime, China), DAPI (C1002, Beyotime, China), RNAiso Plus (9109, TaKaRa, Japan). THUNDERBIRD SYBR qPCR Mix (QPS-201, TOYOBO, Japan), Premix Ex Taq^™^ (Probe qPCR; R390B, Takara, Japan), HiScript II QRT SuperMix for qPCR (gDNA wiper; R223-01, Vazyme, CHINA), X-tremeGENE siRNA Transfection Reagent (04476115001-1ml, Roche, Switzerland), Polyjet *in vitro* DNA transfection reagent (SL100688, Signagen, USA), and TransIT-X2^®^ Dynamic Delivery System (MIR6000, Mirusbio, USA) were used.

### Construction of plasmids

TRIM25 (GenBank accession number: NM_001318458.1) was amplified from the cDNA of DF1 cells via RT-qPCR [51] and inserted into the pCAGGS plasmid with a Flag at the C-terminus, for use in the research experiments. To detect the interaction between TRIM25 and the viral proteins of IBDV, *VP1*, *VP2*, *VP3*, *VP4*, and *VP5* were amplified from the cDNA reverse transcribed from IBDV mRNA and inserted into the pCAGGS plasmid with an HA tag at the N-terminus. To further determine the interaction between TRIM25 and VP3, these plasmids (pMyc-TRIM25, pFlag-VP3, and pMyc-VP3) were constructed and had Flag or Myc tags fused to their 3’ ends.

pMyc-TRIM25DelRING (lacking RING domain) was inserted into the pCAGGS plasmid with an Myc tag fused to the 3’ ends to determine the functional domain of TRIM25 in the ubiquitination of VP3 and in IBDV replication. A schematic diagram of the structural domain of TRIM25 is shown in Fig 6E.

The strategy for constructing the ubiquitination plasmids is shown in Fig 6B. pHA-Ub (WT) was purchased from Genscript (China). The other seven pHA-Ub mutant plasmids (K6, K11, K27, K29, K33, K48, K63) were constructed using site-directed mutagenesis with a strategy in which all lysines (K) were substituted by arginine (R) except for the targeted lysine. The pHA-Ub (K27R) plasmid was constructed using site-directed mutagenesis by only substituting the lysine at position 27 with arginine.

To further demonstrate the ubiquitination site of VP3, six VP3 mutants (pFlag-VP3K760R, pFlag-VP3K832R, pFlag-VP3K854R, pFlag-VP3K889R, pFlag-VP3K912R and pFlag-VP3K963R) were constructed by replacing the targeted lysine with arginine by using site-directed mutagenesis at potential ubiquitination sites shown in Fig 7A.

### IBDV infection

5×10^5^ DF1 cells/DT40 cells were seeded into the plates. When the cells grew to more than 90% adherence and were infected with appropriately diluted viruses (DF-1, 0.1 or MOI; DT40, 5 MOI) for 1.5 h at 38.5 °C in 5% CO_2_. Subsequently, virus cultures were removed, and cells were then washed thrice with D-Hanks or PBS and maintained with DMEM containing 5% FBS for 12, 24, 36, and 48h, respectively. Furthermore, DF-1 cells or TRIM25KO DF-1 cell lines were infected with IBDV Gt strain at an MOI of 0.01 to identify the regulatory role of TRIM25 in IBDV replication according to the manufacturer’s instructions.

### RNA sequencing (RNA-seq)

The DF-1 cells were infected with the IBDV Gt virus at an MOI of 0.01, and the infected and non-infected cells were collected at 24 h p.t. to explore the host factors involved in the process of IBDV infection. After 3,000×*g* centrifugation, the cells were preserved with 1 mL Trizol (TAKARA, Japan) for RNA extraction and subsequent RNA sequencing. Three parallel experiments were carried out.

### Overexpression and RNA interference

To evaluate the influence of TRIM25 on IBDV replication, we detected the replication ability of IBDV by overexpression and RNA interference. For overexpression, DF-1 cells were seeded in 6-well plates and transfected with 2 μg pFlag-TRIM25, pMyc-TRIM25, pMyc-TRIM25DelRING or pCAGGS plasmids, respectively using TransIT-X2^®^ dynamic delivery system, according to the manufacturer’s instructions. After cultured for 24 h, the cells were infected with IBDV Gt strain at an MOI of 0.01. Subsequently, the supernatants and cell cultures were collected at 24 and 48 h p.i., respectively. The relative mRNA expression levels of IBDV *VP5* were detected via RT-qPCR, and the expression levels of VP3 protein of IBDV were detected by Western blotting. Besides, the released viral titers were detected using a TCID_50_ assay [58].

For RNAi, three siRNAs (siTRIM25-1, siTRIM25-2, and siTRIM25-3) specifically targeting the chTRIM25 mRNA were designed by Genechem Company (China). The siRNA sequences used in the experiments were as follows: siTRIM25-1 (sense, 5′-CCAGGUUAGCGGUGAACAATT-3′), siTRIM25-2 (sense, 5′-GCAACACUCUGUUUGGAACTT-3′), siTRIM25-3 (sense, 5′-CCAUGCAAUCUCCUGCUUUTT-3′), and siSc. (sense, 5′-UUCUCCGAACGUGUCACGUTT-3′). First, the three siRNAs were separately co-transfected with the pFlag-TRIM25 plasmids in HEK293T cells by using siRNA transfection reagent according to the manufacturer’s instructions. After incubated for 36 h, the cells were collected to detect the exogenous knockdown levels of TRIM25 by Western blotting. Subsequently, the 1μg siRNA with high knockdown efficiency was transfected into DF-1 cells, and then the cells were collected to detect the endogenous knockdown levels of TRIM25 by RT-qPCR at 24 h p.t. To further evaluate the influence of the TRIM25 on IBDV replication, the siRNA with optimal knockdown efficiency was transfected into DF-1 cells seeded in 6-well plates according to the manufacturer’s instructions. After 24 h, the cells were infected with IBDV Gt strain at an MOI of 0.01and harvested for further analysis at 24 and 48 h p.i., respectively. The relative mRNA levels of IBDV genome in cells were detected via RT-qPCR and the expression levels of VP3 protein of IBDV of cells were detected by Western blotting. Besides, the released viral titers of supernatant were detected using a TCID_50_ assay [58].

### RT-qPCR

Total RNA from the indicated cells that underwent different treatments was extracted using RNAiso Plus (TARAKA) and reverse transcribed into cDNA using reverse transcriptase (Vazyme). To detect the relative RNA quantities changed fold of target genes (TRIM25, *VP3*, and *VP5*), the relative RT-qPCR was performed using the SYBR qPCR Mix (TOYOBO) with a Light Cycler 480 II system (Roche) according to the manufacturer’s instructions. RT-qPCR was performed using the following cycling conditions: 95 °C for 1 min for initial denaturation, followed by 40 cycles of 95 °C for 15 s for denaturation, 60 °C for 1 min, and collection of PCR product signals. The results were analyzed using the ΔΔCt method [59].

The viral loads of IBDV in infected cells were detected using Premix Ex Taq^™^ (Probe qPCR; TAKARA) with Applied Biosystems 7500. The RT-qPCR was performed using the following cycling conditions: one cycle at 48 °C for 30 min and 95 °C for 20s, following by 40 cycles of 95 °C for 3 s and 60 °C for 30 s. Specific primers and TaqMan probes for chicken actin and IBDV *VP5* were synthesized by Invitrogen (China). Specific primers for chicken TRIM25 were designed and synthesized by Comate Bioscience Co., Ltd.

### Western blotting

Cell samples were harvested under different conditions. The samples boiled with 5×SDS loading buffer (P0015L, Beyotime) for 10 min were separated on 10% SDS-PAGE gels and transferred onto a nitrocellulose membrane (Hybond-C Super; GE Healthcare, Piscataway, NJ). All experiments were conducted at room temperature. First, the membrane was blocked in 5% (w/v) skim milk for 1.5 h and incubated with the monoclonal or polyclonal antibodies for 1.5 h. Subsequently, after being washed thrice (10 min each) with PBST, the membrane was incubated with IRDye^®^680RD goat anti-rabbit IgG (H+L) or IRDye 800CW goat anti-mouse IgG(H+L) antibody for 1 h. Finally, the membrane blots were scanned using an Odyssey Infrared Imaging System (Li-Cor Biosciences) for further analysis.

### TCID_50_ titration

CEFs were used to titrate the released infectious virus in different assays. The Infected cell supernatants were harvested at 24 and 48 h p.i., and the titers of supernatants were determined in terms of 50% tissue infection dose (TCID_50_)/100 μL by using the Reed-Muench method [58].

### Generation of TRIM25 knockout(KO) DF-1 cell line

TRIM25KO DF-1 cells line were built depending on the CRISPR/Cas9 method [60]. Firstly, the gRNA targeting TRIM25 mRNA sites (TRIM25: CATCTACTGCGACAGTTGTC) was designed by E-CRISP. Then, the DNA fragments containing the U6 promoter, target RNA sites, and gRNA scaffold were fused and inserted into the pMD18-T vector (TAKARA, Japan). The plasmid was determined by sequencing and extracted with the QIAfilter Plasmid Midi Kit (Qiagen, Germany).

DF-1 cells were seeded in the 6-well plates and cultured to more than 90% adherence. Cells were co-transfected with 1 μg pMJ920 (Addegene: #42234) plasmid and 1 μg plasmid containing gRNA per well (6-well plate) using TransIT-X2^®^ dynamic delivery system according to the manufacturer’s instructions. After 48 h, the positive cells showing green fluorescence were sorted into 12-well plates using flow cytometry. The collected cells were maintained in the growth state, and the cells were diluted to be seeded into the 96-well plates with serial dilutions to obtain a single-cell-derived colony. Approximately two weeks later, genomic DNA was extracted from monoclonal DF-1 cells and determined by sequence analysis and RT-qPCR.

### Cell viability assay

4×10^4^ DF-1 (WT or TRIM25KO) cells were seeded into 96-well plates. Cells were cultured for 6–8 h and were added with 10 μL CCK-8 solution using the cell counting Kit 8 (CCK-8) (Dojindo) according to the manufacturer’s instructions to assess the cell ability. After 2 h, the absorbance of cells was measured at OD450 nm.

### Co-IP

DF-1 cells were seeded in 6-well plates and transfected with the indicated plasmids. Then, the cells were washed thrice with ice-cold PBS and lysed in 500 μL Western blotting and IP lysis buffers (P0013, Beyotime, China) for 30 min. After 12,000×*g* centrifugation, the supernatants of cell lysates were incubated with 1 μg anti-Flag mouse monoclonal antibody (mAb) or control mouse IgG for 6–8h or overnight. Afterward, 40 μL protein A/G agarose (A10001, Abmart) was added to the lysate mixture for 6–8h. The beads were collected by centrifugation at 3,000 ×*g* for 5 min at 4 °C and washed five times with ice-cold PBS. To identify the viral proteins related to TRIM25, 2 μg pHA-VP1, pHA-VP2, pHA-VP3, pHA-VP4, or pHA-VP5 and 2 μg pFlag-TRIM25 were co-transfected, respectively, and the search was then conducted following the above instruction. Furthermore, the relationship between TRIM25 and VP3 was detected in both directions by co-transfecting pFlag-plasmids and pMyc-plasmids.

### Confocal microscopy and IFA

DF-1 cells were co-transfected with pFlag-TRIM25 and/or pHA-VP3 for 24 h. The cells were washed thrice with ice-cold PBS and then fixed in 4% (v/v) paraformaldehyde for 30 min at 24 h p.t. at room temperature. After being washed thrice with ice-cold PBS, the cells were blocked with 5% (w/v) bovine serum albumin for 1.5 h at 37 °C. Similarly, after being washed thrice with ice-cold PBS, the cells were incubated with anti-HA mAb produced in mice and anti-TRIM25 polyclonal antibody produced in rabbits for 1.5 h at 37 °C. After being washed three times with ice-cold PBS, the cells were incubated with Alexa Fluor 488 Goat anti-rabbit IgG (H+L) and Alexa Fluor 546 goat anti-mouse IgG (H+L) for 1 h. Finally, the cells were stained with DAPI for 10 min at room temperature and examined using a Leica SP2 confocal system (Leica Microsystems, Wetzlar, Germany) to identify the relationship between TRIM25 and VP3.

For IFA, DF-1 cells were infected with rmGt and rGt-VP3K854R at an MOI of 0.01 and then were treated according to the protocols of confocal microscopy. The cells were incubated with anti-VP2/VP3 monoclonal antibodies for 1.5 h at 37 °C. After being washed three times with ice-cold PBS, the cells were incubated with FITC and Alexa Fluor 546 goat anti-mouse IgG (H+L) for 1 h. Finally, the cells were stained with DAPI for 10 min at room temperature and to determine the expression of viral proteins.

### Ubiquitination assay

To analyze the influence of TRIM25 on the ubiquitination of VP3, pFlag-VP3 was co-transfected with pHA-Ub and pMyc-TRIM25 or empty vector plasmids into HEK293T cells. After being cultured for 48 h, the cells were harvested in NP40 buffer containing protease inhibitors with 1mM phenylmethanesulfonyl fluoride (PMSF) and incubated for 30 min on ice. The lysates were centrifuged and immunoprecipitated with 2 μg anti-Flag mAb produced in mice and analyzed via immunoblotting analysis with anti-HA, anti-TRIM25, and anti-Flag antibodies. Similarly, HEK293T cells were co-transfected with the pFlag-VP3 plasmid and the pHA-Ub, pMyc-TRIM25, and pMyc-TRIM25DelRING plasmids to identify the functional domain of TRIM25 related to ubiquitination.

To analyze the type of ubiquitination that VP3 underwent, pFlag-VP3 and pMyc-TRIM25 plasmids were co-transfected with different pHA-Ub (WT, K6, K11, K27, K29, K33, K48, K63, or K27R) plasmids into HEK293T cells, respectively. According to the procedures mentioned, the lysate were centrifuged and immuno-precipitated (IP) with 2 μg anti-Flag mAb produced in mice and analyzed by Western blotting with anti-HA, anti-TRIM25, and anti-Flag antibodies.

To analyze the key ubiquitination site of VP3, different pFlag-VP3 plasmids (pFlag-VP3K760R, K832R, K854R, K889R, K912R, or K963R) were co-transfected with the pHA-Ub and pMyc-TRIM25 plasmids into HEK293T cells. According to the procedures mentioned above, the lysates were centrifuged and immunoprecipitated with 2 μg anti-Flag mAb produced in mice and analyzed by Western blotting with anti-HA, anti-TRIM25 and anti-Flag antibodies.

### Reverse genetics

The IBDV was rescued using the RNA polymerase II system [33]. The pCAGGGtAHRT and pCAGGGtBHRT plasmids were constructed and preserved in our laboratory [33]. The mutant plasmid pCAGGGtA-VP3K854RHRT was constructed using a direct mutation assay. The pCAGGGtAHRT or pCAGGGtA-VP3K854RHRT plasmids were co-transfected with pCAGGGtBHRT plasmids into DF-1 cells, respectively. At 72 h p.t., after freezing and thawing three times, the cells were harvested by centrifugation at 300×g for 5 min at 4 °C. The lysates were used to blindly passage in CEFs until the cytopathic effect was obvious. The viruses rescued successfully ere named rmGt and rGt-VP3K854R.

### Virus growth curve

WT (rmGt) and mutant IBDV (rGt-VP3K854R) viruses, CEFs were infected with two viruses at an MOI of 0.01 and subsequently were performed using the procedures mentioned above at 12, 24, 36, 48, 60, and 72 h. The released viral titers of the collected samples were detected using the TCID_50_ assay. Then the viral growth curve was drawn based on the titers of supernatants at different infection time points.

### Animal experiments

Thirty-six 3-week-old SPF chickens were randomly divided into three groups and were infected with viruses at 10^5.8^TCID_50_/200 μL or 200 μL PBS, respectively, to check the replication characteristics of the WT (rmGt) and mutant IBDV (rGt-VP3K854R) viruses *in vivo*. To compare the replication ability of rmGt and rGt-VP3K854R viruses, nine bursae (three bursae per group) were collected at 3, 5, 7, and 14 days p.i, respectively. The viral loads of collected bursae were detected via RT-qPCR using a TaqMan probe targeting the IBDV VP5 gene.

The death of chickens, the atrophy of bursae and the pathological injury of bursae were the evaluation index of virulence of IBDV. The symptoms of chickens were counted and the bursa and body weights of all chickens were determined to check the virulence of the two viruses. The bursa: body weight index (BBIX) was calculated along with the standard deviation [BBIX = (bursa: body weight ratios in the virus-infected group)/(bursa: body weight ratios in the blank group)]. Bursae with BBIX <0.70 were considered atrophied [37]. Simultaneously, bursae from different groups were fixed by immersion in 10% neutral buffered formalin and stained with hematoxylin and eosin for further histopathological examination. All animal experiments were approved by the Committee on the Ethics of Animal Experiments at the Harbin Veterinary Research Institute (Harbin, China), Chinese Academy of Agricultural Sciences, and performed following the guidelines for experimental animals of the Ministry of Science and Technology (Beijing, China).

### Statistical analysis

All experiments were performed in triplicate on the same plate. Data are presented as the mean ± standard deviation (SD). In some experiments, the significance of the variability between different groups was determined by two-way ANOVA using the GraphPad Prism software (version 8.0). A *p*-value <0.05 was considered statistically significant and marked with an asterisk (*). In other experiments, statistical analyses were performed with the unpaired t-test, and *p* <0.05 was considered significant.

## Supporting information

S1 Fig Relative expression of chicken TRIMs upon IBDV infection. The RNA-seq analysis of TRIMs expression levels upon IBDV infection compared to that of non-infected in DF-1 cells at 24 h p.i.
(TIF)
