# Supplementary figures and images for: TRIM25 inhibits infectious bursal disease virus replication by targeting VP3 for ubiquitination and degradation
Source: PLoS Pathog. 2021 Sep 13;17(9):e1009900. doi: 10.1371/journal.ppat.1009900 (PMC8459960; doi:10.1371/journal.ppat.1009900)

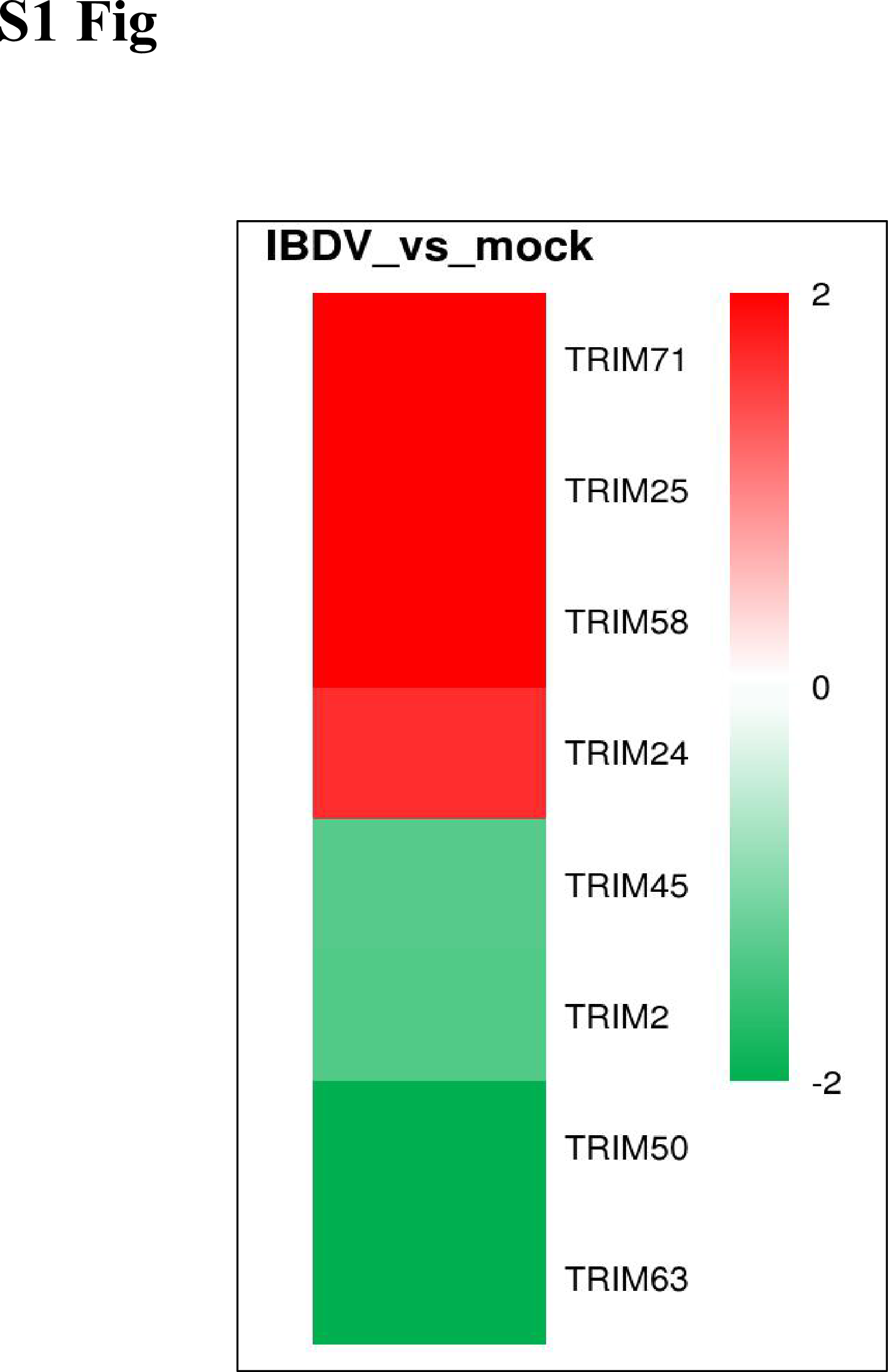

Supplement: S1 Fig — The RNA-seq analysis of TRIMs expression levels upon IBDV infection compared to that of non-infected in DF-1 cells at 24 h p.i. (TIF) [file ppat.1009900.s001.tif]
